# Supplementary material for: Comprehensive Transcriptome Analysis Reveals the Distinct Gene Expression Patterns of Tumor Microenvironment in HPV-Associated and HPV-Non Associated Tonsillar Squamous Cell Carcinoma
Source: Cancers (Basel). 2023 Nov 23;15(23):5548. doi: 10.3390/cancers15235548 (PMC10705173; doi:10.3390/cancers15235548)
Supplement: Supplementary file 1 [file cancers-15-05548-s001.zip › cancers-2641174-supplementary.pdf]

Supplementary Materials: Comprehensive Transcriptome Analysis Reveals the Distinct Gene Expression Patterns of Tumor Microenvironment in HPV-Associated and HPV-Non Associated Tonsillar Squamous Cell Carcinoma

Reham M. Alahmadi, Najat Marraiki, Mohammed Alswayyed, Hatim A. Khoja, Abdullah E. Al-Anazi, Rawan M. Alahmadi, Meshael M. Alkusayer, Bandar Alosaimi and Maaweya Awadalla

Table S1. UniGene, GenBank, Symbol and description of Genes.

UniGene	GenBank	Symbol	Description
Hs.431048	NM_005157	ABL1	C-abl oncogene 1, non-receptor tyrosine kinase
Hs.525622	NM_005163	AKT1	V-akt murine thymoma viral oncogene homolog 1
Hs.158932	NM_000038	APC	Adenomatous polyposis coli
Hs.367437	NM_000051	ATM	Ataxia telangiectasia mutated
Hs.624291	NM_004324	BAX	BCL2-associated X protein
Hs.517461	NM_004327	BCR	Breakpoint cluster region
Hs.34012	NM_000059	BRCA2	Breast cancer 2, early onset
Hs.599762	NM_001228	CASP8	Caspase 8, apoptosis-related cysteine peptidase
Hs.461086	NM_004360	CDH1	Cadherin 1, type 1, E-cadherin (epithelial)
Hs.370771	NM_000389	CDKN1A	Cyclin-dependent kinase inhibitor 1A (p21, Cip1)
Hs.512599	NM_000077	CDKN2A	Cyclin-dependent kinase inhibitor 2A (melanoma, p16, inhibits CDK4)
Hs.72901	NM_004936	CDKN2B	Cyclin-dependent kinase inhibitor 2B (p15, inhibits CDK4)
Hs.654393	NM_005225	E2F1	E2F transcription factor 1
Hs.181128	NM_005229	ELK1	ELK1, member of ETS oncogene family
Hs.208124	NM_000125	ESR1	Estrogen receptor 1
Hs.369438	NM_005238	ETS1	V-ets erythroblastosis virus E26 oncogene homolog 1 (avian)
Hs.728789	NM_005252	FOS	FBJ murine osteosarcoma viral oncogene homolog
Hs.396530	NM_000601	HGF	Hepatocyte growth factor (hepapoietin A; scatter factor)
Hs.37003	NM_005343	HRAS	V-Ha-ras Harvey rat sarcoma viral oncogene homolog
Hs.656213	NM_004972	JAK2	Janus kinase 2
Hs.479754	NM_000222	KIT	V-kit Hardy-Zuckerman 4 feline sarcoma viral oncogene homolog
Hs.632486	NM_021960	MCL1	Myeloid cell leukemia sequence 1 (BCL2-related)
Hs.484551	NM_002392	MDM2	Mdm2 p53 binding protein homolog (mouse)
Hs.533432	NM_005372	MOS	V-mos Moloney murine sarcoma viral oncogene homolog
Hs.654446	NM_005375	MYB	V-myb myeloblastosis viral oncogene homolog (avian)
Hs.202453	NM_002467	MYC	V-myc myelocytomatosis viral oncogene homolog (avian)
Hs.25960	NM_005378	MYCN	V-myc myelocytomatosis viral related oncogene, neuroblastoma derived (avian)
Hs.113577	NM_000267	NF1	Neurofibromin 1
Hs.81328	NM_020529	NFKBIA	Nuclear factor of kappa light polypeptide gene enhancer in B-cells inhibitor, alpha
Hs.486502	NM_002524	NRAS	Neuroblastoma RAS viral (v-ras) oncogene homolog
Hs.175343	NM_002645	PIK3C2A	Phosphoinositide-3-kinase, class 2, alpha polypeptide
Hs.553498	NM_006218	PIK3CA	Phosphoinositide-3-kinase, catalytic, alpha polypeptide
Hs.526464	NM_033238	PML	Promyelocytic leukemia
Hs.531704	NM_002737	PRKCA	Protein kinase C, alpha
Hs.159130	NM_002880	RAF1	V-raf-1 murine leukemia viral oncogene homolog 1
Hs.654583	NM_000964	RARA	Retinoic acid receptor, alpha
Hs.476270	NM_007182	RASSF1	Ras association (RalGDS/AF-6) domain family member 1
Hs.408528	NM_000321	RB1	Retinoblastoma 1
Hs.631886	NM_002908	REL	V-rel reticuloendotheliosis viral oncogene homolog (avian)
Hs.350321	NM_020630	RET	Ret proto-oncogene
Hs.149261	NM_001754	RUNX1	Runt-related transcription factor 1
Hs.654444	NM_002961	S100A4	S100 calcium binding protein A4
Hs.55279	NM_002639	SERPINB5 Maspin	Serpin peptidase inhibitor, clade B (ovalbumin), member 5
Hs.515005	NM_000455	STK11	Serine/threonine kinase 11
Hs.370854	NM_000368	TSC1	Tuberous sclerosis 1
Hs.517792	NM_000551	VHL	Von Hippel-Lindau tumor suppressor
Hs.461453	NM_016373	WWOX	WW domain containing oxidoreductase
Hs.98493	NM_006297	XRCC1	X-ray repair complementing defective repair in Chinese hamster cells 1
Hs.471751	NM_020311	ACKR3	Chemokine (C-X-C motif) receptor 7

Hs.150749	NM_000633	BCL2	B-cell CLL/lymphoma 2
Hs.143961	NM_002988	CCL18	Chemokine (C-C motif) ligand 18 (pulmonary and activation-regulated)
Hs.75498	NM_004591	CCL20	Chemokine (C-C motif) ligand 20
Hs.534347	NM_002990	CCL22	Chemokine (C-C motif) ligand 22
Hs.75703	NM_002984	CCL4	Chemokine (C-C motif) ligand 4
Hs.514821	NM_002985	CCL5	Chemokine (C-C motif) ligand 5
Hs.705362	NM_001123396	CCR2	Chemokine (C-C motif) receptor 2
Hs.370036	NM_001838	CCR7	Chemokine (C-C motif) receptor 7
Hs.521989	NM_014143	CD274	CD274 molecule
Hs.1349	NM_000758	CSF2	Colony stimulating factor 2 (granulocyte-macrophage)
Hs.2233	NM_000759	CSF3	Colony stimulating factor 3 (granulocyte)
Hs.247824	NM_005214	CTLA4	Cytotoxic T-lymphocyte-associated protein 4
Hs.624	NM_000584	CXCL8	Chemokine (C-X-C motif) ligand 8 (IL8)
Hs.632586	NM_001565	CXCL10	Chemokine (C-X-C motif) ligand 10
Hs.522891	NM_000609	CXCL12	Chemokine (C-X-C motif) ligand 12
Hs.77367	NM_002416	CXCL9	Chemokine (C-X-C motif) ligand 9
Hs.846	NM_001557	CXCR2	Chemokine (C-X-C motif) receptor 2
Hs.605083	NM_005228	EGFR	Epidermal growth factor receptor
Hs.247700	NM_014009	FOXP3	Forkhead box P3
Hs.62661	NM_002053	GBP1	Guanylate binding protein 1, interferon-inducible
Hs.1051	NM_004131	GZMB	Granzyme B (granzyme 2, cytotoxic T-lymphocyte-associated serine esterase 1)
Hs.719495	NM_001530	HIF1A	Hypoxia inducible factor 1, alpha subunit (basic helix-loop-helix transcription factor)
Hs.713441	NM_002116	HLA-A	Major histocompatibility complex, class I, A
Hs.77961	NM_002117	HLA-C	Major histocompatibility complex, class I, C
Hs.856	NM_000619	IFNG	Interferon, gamma
Hs.674	NM_002187	IL12B	Interleukin 12B (natural killer cell stimulatory factor 2, cytotoxic lymphocyte maturation factor 2, p40)
Hs.845	NM_002188	IL13	Interleukin 13
Hs.602618	NM_000585	IL15	Interleukin 15
Hs.1722	NM_000575	IL1A	Interleukin 1, alpha
Hs.591803	NM_016584	IL23A	Interleukin 23, alpha subunit p19
Hs.73917	NM_000589	IL4	Interleukin 4
Hs.654458	NM_000600	IL6	Interleukin 6 (interferon, beta 2)
Hs.407995	NM_002415	MIF	Macrophage migration inhibitory factor (glycosylation-inhibiting factor)
Hs.202453	NM_002467	MYC	V-myc myelocytomatosis viral oncogene homolog (avian)
Hs.82116	NM_002468	MYD88	Myeloid differentiation primary response gene (88)
Hs.313	NM_000582	SPP1	Secreted phosphoprotein 1
Hs.519033	NM_003264	TLR2	Toll-like receptor 2
Hs.241570	NM_000594	TNF	Tumor necrosis factor
Hs.73793	NM_003376	VEGFA	Vascular endothelial growth factor A

Table S2. PCR primers used for HPV genotyping and oncogenes integration detection.

Primer	Specificity	Sequence (5′ to 3′)
**HPV16**	F16E7	TCAGAGGAGGAGGATGAAATAGA
	R16E7	GCACAACCGAAGCGTAGA
	F16E6	AATGTTTCAGGACCCACAGG
	R16E6	GTTGCTTGCAGTACACACATTC
**HPV18**	F18E1	CATTTTGTGAACAGGCAGAGC
	R18E1	ACTTGTGCATCATTGTGGACC
	F18E6	ACCCTACAAGCTACCTGATCT
	R18E6	ACCTCTGTAAGTTCCAATACTGTC
	F18E7	AATTCCGGTTGACCTTCTATGT
	R18E7	GGCTGGTAAATGTTGATGAT
**HPV31**	F31E6	ACGATTCCACAACATAGGAGGA
	R31E6	TACACTTGGGTTTCAGTACGAGGT
**HPV33**	F33L1	CGTCGCAGGCGTAAACG
	R33/etcI.1	ACAGGAGGCAGGTACAC
**HPV35**	F35E4	GCCTGCTCCGTGGGC
	R35E4	GCACTGAGTCGCACTCGC
**HPV39**	F39E7	CGAGCAATTAGGAGAGTCAGAGG
	R39E7	TGTGTGACGCTGTGGTTCAT
**HPV45**	F18E1	CATTTTGTGAACAGGCAGAGC
	R45E1	CAACACCTGTGCATCATTCTGA

**HPV58**	F58L1	GCGTCGCAGACGTAAACG
	R33/etcI.1	ACAGGAGGCAGGTACAC
**HMBS**	F HMBS	GCCTGCAGTTTGAAATCAGTG
	R HMBS	CGGGACGGGCTTTAGCTA
**GAPDH**	F GAPDH	GTATTGGGCGCCTGGTCACC
	R GAPDH	CGCTCCTGGAAGATGGTGATGG
